# Examining the Relationship Between Basic Nursing Skills, Hand Function, and Anxiety Levels in First‐Year Nursing Students: A Correlational Study

**DOI:** 10.1111/jep.70191

**Published:** 2025-06-29

**Authors:** Yılmaz Coşkun Ela, Yalçın Irmak Aylin, Şendir Merdiye

**Affiliations:** ^1^ Department of Nursing Tekirdag, Faculty of Health Sciences Tekirdağ Namık Kemal University Tekirdag Turkey; ^2^ Mekteb‐i Tıbbiyye‐i Şahane (Haydarpaşa) Complex, Hamidiye Faculty of Nursing University of Health Sciences Istanbul Turkey

**Keywords:** anxiety, hand function, nursing, Purdue Pegboard Test, skill

## Abstract

**Aim:**

The aim of this study was to determine the relationship between basic nursing skills, hand functions and anxiety levels of nursing students.

**Background:**

It is vital that basic nursing skills are achieved in the fastest and most accurate way in nursing.

**Design:**

This study was conducted with cross‐sectional correlational design.

**Methods:**

The sample of study consisted of freshman nursing students (*N *= 123). The data were obtained by ‘Student Information Form’, ‘State‐Trait Anxiety Inventory’, ‘Basic Nursing Skills Forms’ and ‘Hand Skill Test’. Descriptive statistics and Pearson's correlation coefficient were used to analyse the data.

**Results:**

The mean age of the students was 19.24 ± 1.31 years and 69.9% were female. 76% (*n *= 95) of them had not experienced any nursing skill before. The students' scores of basic nursing skills were above average. State and Trait Anxiety Inventory total scores were 44.71 ± 6.57 and 39.78 ± 9.82, respectively. 93.6% (*n* = 117) of the students had right dominant hand. Significant low‐to‐moderate correlations were found between left hand fine motor skill score and nasogastric drug administration skill (*r* = 0.197), both hand fine motor skill scores and blood collection skill (*r* = 0.191), assembly fine motor skill score and sterile glove wearing (*r* = 0.200), and subcutaneous injection skill (*r* = 0.277). However, no significant correlation was found between hand function and anxiety levels.

**Conclusions:**

In this study, a relationship was found between hand function and some basic skills. It is recommended that hand skills of nursing students should be evaluated multidimensionally with different measurement tools and similar studies should be planned in groups at different levels of nursing education and in larger samples.

## 1 Introduction

Nursing is a branch of art and science in which special knowledge and skills must be applied to ensure holistic and effective care [1]. In nursing education, it is essential to adopt a curriculum that integrates knowledge, skills, and professional attitudes [2]. Nursing students are expected to acquire numerous psychomotor skills that address the care needs of both healthy and sick individuals and represent the core of the profession [3, 4, 5].

Psychomotor skills, often referred to as basic clinical or technical skills, include attention‐requiring procedures (e.g., intravenous injection), manipulative procedures (e.g., nasal aspiration, wound care), and movement‐based tasks (e.g., CPR, patient care techniques) [6, 7, 8]. These are coordinated muscle activities governed by conscious mental effort and are recognised as essential competencies for newly graduated nurses [9].

Manual skill is a key indicator of hand‐related psychomotor function and involves the integration of motor and sensory systems to perform actions such as manipulation, fixation, and grasping [10, 11, 12]. Fine motor dexterity is particularly critical in nursing procedures that demand precision, such as intravenous catheter insertion, wound dressing, medication administration, and surgical suturing. The Purdue Pegboard Test (Lafayette Instruments, Lafayette, IN) is frequently recommended by researchers due to its reliability in evaluating fine motor skills and its sensitivity in detecting subtle changes, particularly in vocational and healthcare‐related applications [13, 14]. As it measures bilateral hand coordination, fine motor control, and manipulative ability under timed conditions, the Purdue Pegboard Test provides an objective means of assessing a student's readiness for skill‐intensive nursing procedures. The ability to perform repetitive, intricate hand movements efficiently is essential to minimising procedural errors and promoting patient safety. In the literature, a decline in manual skills has been linked to impaired daily functioning, reduced work efficiency, trauma, neurological disorders, and cognitive impairment [15, 16]. Many studies emphasise that young individuals and students must possess high‐level manual dexterity not only to succeed in daily life activities but also to effectively apply vocational knowledge and skills [17, 18, 19]. Manual skills are considered a key parameter in the acquisition and application of vocational competencies [20]. In this context, the Purdue Pegboard Test serves as a meaningful proxy for evaluating a student's potential to perform critical manual tasks required in safe and effective clinical nursing practice.

Hand skills of nursing students are undoubtedly important in providing safe and effective nursing care [8]. It is a desirable competence for nursing students to be able to use their hands and fingers skilfully in the continuation of care and at the same time to maintain effective communication with healthy/sick individuals. Moreover, psychomotor skills are essential in nursing to evaluate competence in nursing skills and to provide the most appropriate and safe care to all patients [7, 21]. Deficiencies in psychomotor skills may lead to procedural errors, medication mishandling, or compromised infection control practices, all of which pose direct risks to patient safety [22]. Thus, the psychomotor competence of nursing students is not only an educational concern but a critical determinant of patient outcomes and safety [23].

There is ongoing debate about the best ways to assess nurses' skills. In a systematic review of 15 research articles, Missen et al. [9] found concern about the clinical and technical skill levels of newly graduated nurses. Similarly, Romyn et al. [24] argued that while theoretical knowledge remains a key component of nursing education, it does not necessarily lead to graduates with strong psychomotor competence. In Hickey's [25] study, 63% of trainers stated that newly graduated nurses needed additional support with technical skills.

While previous studies have independently explored the roles of manual skills and psychological factors such as anxiety, few have explored their combined and interacting influence on psychomotor skill acquisition in nursing students. Most existing research addresses these variables in isolation, overlooking their possible interaction in shaping clinical readiness [8, 20]. Understanding the conceptual relationships among these variables is essential, as manual dexterity directly contributes to the successful execution of clinical procedures, while anxiety may impair both cognitive concentration and fine motor control [26, 27]. Although factors such as age, gender, dominant hand, and glove use have been associated with hand skills, the potential impact of anxiety on both hand function and psychomotor competence remains largely underexplored. Considering that anxiety can impair both motor performance and cognitive processes, it is important to investigate its role in skill development during nursing education. Another important gap relates to the student selection process. While university entrance exams assess theoretical knowledge, they do not evaluate students' manual dexterity, which may be critical for mastering practical nursing skills. This raises questions about whether the initial hand skill level of students influences their competence at the end of their undergraduate education. To address these gaps, the present study integrates manual dexterity, anxiety, and basic nursing skills within a single framework. The findings, in alignment with the World Health Organisation's [23] emphasis on competency‐based nursing education, may contribute to the development of more comprehensive and practice‐oriented nursing curricula on both national and international levels.

### 1.1 Aim

In this study, it was aimed to examine the relationship between basic nursing skills, hand function and anxiety levels of nursing students. By identifying these relationships, the study seeks to contribute to the enhancement of psychomotor skill training in nursing education and support the development of targeted educational strategies to improve student readiness for clinical practice.

## 2 Methods

### 2.1 Study Design and Sample

The study, which was a cross‐sectional correlational type, was carried out with first‐year students who voluntarily participated in the study (*N* = 139) studying in the nursing department of a state university in Turkey. Students with physical and sensory (visual, hearing) disabilities and students with a history of traumatic or atraumatic upper extremity disorders in the past year were not included in the study. Between 1 and 30 June 2023, when the data were collected, 16 students dropped out of the study due to reasons such as absenteeism or wanting to leave the study. The effect sizes obtained for postoperative strength based on the both hands and assembly hand skills of female and male students were 0.547 and 0.632, respectively, with a power of 95% for both.

### 2.2 Data Collection Tools

The data were obtained by ‘Student Information Form’, ‘State‐Trait Anxiety Inventory’, ‘Basic Nursing Skills Forms’ and ‘Hand Skill Test’.

#### 2.2.1 Student Information Form (SIF)

The form, which was created by the researchers by examining the relevant literature, includes a total of 14 questions about the students' socio‐demographic characteristics (age, gender, chronic disease status, etc.), hobbies (sports, music, etc.) and nursing skill experiences.

#### 2.2.2 State‐Trait Anxiety Inventory

It was developed by Spielberger et al. in 1970 and its Turkish validity and reliability study was conducted by Öner and Le Compte [28]. It is a 2‐part Likert‐type scale that measures state and trait anxiety levels separately with 20 questions. Direct statements express negative emotions and reverse items express positive emotions. Questions 1, 2, 5, 8, 10, 11, 15, 16, 19 and 20 are reverse items for the State Anxiety Inventory (SAI); questions 21, 26, 27, 30, 33, 36 and 39 are reverse items for the Trait Anxiety Inventory (TAI). A constant value of 50 is added to the total score for the TAI and a value of 35 is added to the total score for the SAI. The last value obtained is the anxiety score of the individual. A low score indicates a low level of anxiety, while a high score indicates a high level of anxiety. The Cronbach Alpha internal consistency reliability coefficient of the scale was found to be between 0.94 and 0.96 for TAI and between 0.83 and 0.87 for SAI. For the sample of this study, the Cronbach Alpha internal consistency reliability coefficient for the TAI was 0.896 and the Cronbach Alpha internal consistency reliability coefficient for the SAI was 0.835.

#### 2.2.3 Basic Nursing Skills Forms

These data collection tools cover the basic nursing skills aimed to be acquired in the ‘Fundamentals of Nursing’ course. In the study, 11 basic nursing skills (hand washing, perineal care for women, special oral care, wearing sterile gloves, blood pressure measurement, intramuscular injection, subcutaneous injection, blood collection, peripheral catheter application, drug preparation using ampoules/vials, nasotracheal aspiration and nasogastric drug administration) taught by the expert researcher were determined. The achievement scores of the students were obtained for each skill assessed by means of existing cheklists. In such cheklists, there are process steps specific to the relevant skill. It makes it easier to give points according to whether the student performs the steps to be applied in the skill or not. In the evaluation, if the student could not perform or skipped any process step, it was marked as ‘0’ point, if the student did it but there was a situation that needed to be improved, it was marked as ‘2.5’ points and when it was realised at the desired level, it was marked as ‘5’ points. All assessments were obtained by the same researcher. Nursing students experienced one skill each week.

#### 2.2.4 Hand Skill Test

Students' manual skills were assessed with the ‘Purdue Pegboard Test’. This test (developed by Lafeyette instruments, model 3202) assesses fine motor skills by emphasising the speed and manipulative ability of the hands and fingers. The test was administered in accordance with the user manual (available at: http://www.limef.com/downloads/MAN-32020A-forpdf-rev0.pdf). Four subtests were conducted in the study: right hand, left hand, both hands and assembly subtest. The one‐handed subtest (right/left) consists of inserting as many pins into the slot as possible within 30 s. The score represents the number of correctly inserted pins. Both hand subtests are limited to 30 s, with the left and right hand being used simultaneously to insert pins into the slots in both columns. The score indicates the number of pairs of pins inserted. To complete the 1‐min Assembly Sub‐Test it is necessary to hold and insert the pins, washers and clamping ring and the two hands are used alternately. The score indicates the number of pins inserted into the slots.

### 2.3 Procedure

The study was conducted in two stages with first year nursing students who agreed to participate in the study: *In the first stage*, the students participating in the study were asked to answer the SIF, TAI and SAI. Afterwards, in a room isolated from noise and crowds where only the student and the researcher would be present, the student was informed about the manual assessment tool, the Hand Skill Test (Purdue pegboard test), and was asked to perform the test once for trial purposes. Four subtests were performed with the Manual Skill Test: right hand subtest, left hand subtest, both hand subtest and assembly subtest. After the start command, a stopwatch was activated and the number of pins inserted into the slots was counted at the end of 30 s with the right/left hand and both hands. In the assembly subtest, the number of pins inserted into the slots with the washers mounted on the pins and the clamping ring was counted for 1 min. When the participants dropped one of the pins during the test, they picked up a new one and continued the test without stopping. When the time of 30 s/1 min, which was evaluated using a stopwatch, expired, the test ended with the stop command and the number of pins placed in the slots was recorded by the researchers.

*In the second stage*, the theoretical part including the subject of the skill was explained to the students in the classroom environment by the expert researcher in the field. After the lecture, students were shown the demonstration of the skill in the skill laboratory. Skill models (intravenous arm model, intramuscular hip model, etc.) were used as educational tools in the demonstration. Students were allocated time to try each skill in groups and their questions were answered. On the next day, all students performed the skills they had learnt on the skill models and were evaluated according to the cheklist. Necessary precautions (light, temperature, noise, etc.) were taken in terms of situations that could affect the student's skill. When the practice of each skill was completed, the student's score was determined between 0 and 100 and recorded. Student feedback was provided verbally immediately after the application. Nursing students practised one skill each week. In each new skill teaching, this procedure was observed as standard. All skill evaluations were carried out by a single expert researcher to ensure assessment consistency and to eliminate potential inter‐rater variability.

### 2.4 Data Analysis

The data obtained in the study were analysed using Windows IBM SPSS Statistics 22 software. Mean, percentage, standard deviation, minimum and maximum values were calculated for descriptive statistics. The relationship between the basic nursing skills of the students and some descriptive characteristics, total scores of the SAI‐TAI and motor skill scores were analysed by Pearson's correlation coefficient. The normality of the data for gender comparisons was assessed using skewness and kurtosis statistics. Subsequently, an independent samples *t*‐test was applied to examine whether there was a significant difference between genders. Significance level was accepted as 0.05.

### 2.5 Ethical Considerations

Research data were collected after the ethical committee (Tekirdağ Namık Kemal University Ethical Committee (Project Data/Number: 2023.112.05.31) and institutional permission were completed. Written and verbal consents of the participants were obtained at the beginning of data collection.

## 3 Results

### 3.1 Students' Descriptive Characteristics and Basic Nursing Skills Test Results

The mean age of the nursing students (*N* = 123) participating in the study was 19.74 ± 1.31, 69.9% were female (*n* = 87). The mean screen time of the students was 4.30 ± 1.91/h on weekdays and 6.73 ± 3.01/h on weekends. 76% of the students (*n *= 95) stated that they had not experienced any nursing skill before. The mean scores of the students for basic nursing skills were 91.14 ± 7.09 for hand washing, 77.72 ± 7.48 for perineal care of women, 90.24 ± 5.50 for special oral care, 92.07 ± 8.66 for wearing sterile gloves, 89.83 ± 6.81 for blood pressure measurement, 88.35 ± 6.41 for intramuscular injection, 89.97 ± 6.56 for subcutaneous injection, 96.03 ± 3.65 for blood collection, 94.68 ± 4.71 for peripheral catheter application, 87.36 ± 8.26 for drug preparation from ampoules/vials, 85.89 ± 8.69 for nasotracheal aspiration and 93.23 ± 3.84 for nasogastric drug administration (Table 1).

**TABLE 1:** Students' descriptive characteristics and basic nursing skills test results (*N* = 123).

Descriptive characteristics	Mean	SD	Min.	Max.
Age	19.74	1.306	18	28
Gender				
Female (*n*/%)	87 (69.9)			
Male (*n*/%)	38 (30.4)			
Screen time (day/week)				
On weekdays	4.30	1.910	0	10
On weekends	6.73	3.012	0	20
Experienced any nursing skill before				
Yes	30 (%24)			
No	95 (%76)			
Basic nursing skills				
Hand washing	91.14	7.09	55	100
Perineal care for women	77.72	7.48	60	100
Special oral care	90.24	5.50	75	100
Wearing sterile gloves	92.07	8.66	60	100
Blood pressure measurement	89.83	6.81	65	100
İntramuscular injection	88.35	6.41	55	97.5
Subcutaneous injection	89.97	6.56	67.5	100
Blood collection	96.03	3.65	80	100
Peripheral catheter application	94.68	4.71	80	100
Drug preparation using ampoules/vials	87.36	8.26	60	100
Nasotracheal aspiration	85.89	8.69	60	100
Nasogastric drug administration	93.23	3.84	77.5	100

### 3.2 Students' Anxiety Scores and Hand Skill Test Results

When the hand function tests of the students were analysed, 93.6% (*n* = 117) had right dominant hand. The mean score of the right hand subtest of fine motor skills was 14.92 ± 1.93, the mean score of the left hand subtest was 13.76 ± 1.75, the mean score of both hand subtests was 11.18 ± 1.66, and the mean score of the assembly subtest was 31.24 ± 6.17. When comparing the mean scores of the male and female students on the hand skill tests, no significant difference was found for the right hand subtest. However, significant differences in favour of female students were found for the left hand, both hands, and assembly subtests (*p *< 0.05). The SAI and TAI total scores of the students who participated in the study were 44.71 ± 6.57 and 39.78 ± 9.82, respectively. When comparing the SAI and TAI total scores of female and male students, no significant difference was found between the genders for the total score (*p *> 0.05) (Table 2).

**TABLE 2:** Students' anxiety scores and hand skill test results (*N* = 123).

Hand skill subtests	Total		Female	Male	Test/*p*
	Mean ± SD	Min.‐max.			
Right hand	14.92 ± 1.93	10–20	15.10 ± 1.93	14.49 ± 1.88	1.639/0.104
Left hand	13.76 ± 1.75	8–17	13.99 ± 1.70	13.24 ± 1.77	2.204/0.029
Both hand	11.18 ± 1.66	12–30	22.91 ± 3.05	21.08 ± 3.62	2.878/0.005
Assembly	31.24 ± 6.17	17–53	32.33 ± 6.27	28.70 ± 5.15	3.091/0.002
SAI total score	44.71 ± 6.57	22–65	45.63 ± 8.57	42.59 ± 7.61	1.859/0.055
TAI total score	39.78 ± 9.82	20–68	39.79 ± 9.83	39.75 ± 9.93	0.018/0.986
Dominant hand					
Right (*n*/%)	117/93.6				
Left (*n*/%)	8/6.4				

### 3.3 Relationship Results Between Students Basic Nursing Skills and Some Descriptive Characteristics, Anxiety and Hand Skill Test Results

When the relationship between the basic nursing skills of the students and the total scores of the SAI‐TAI and the hand skill test scores was analysed, a significant positive low relationship was found between the left hand skill score and the nasogastric drug administration skill (r = 0.197, *p *< 0.05). A significant positive low correlation was found between both hand skill scores and blood collection skill scores (r = 0.191, *p* < 0.05). A significant positive low correlation was found between the assembly skill score and the ability to wear sterile gloves (r = 0.200, *p* < 0.05) and subcutaneous injection skill (r = 0.277, *p* < 0.01). A significant negative low correlation was found between TAI total score and nasotracheal aspiration skill (r = −0,182, *p* < 0.05) (Table 3).

**TABLE 3:** Relationship results between students' basic nursing skills and some descriptive characteristics, anxiety and hand function test results.

	Hand washing	Perineal care for women	Special oral care	Wearing sterile gloves	Blood pressure measurement	IM injection	SC injection	Blood collection	Peripheral catheter application	Drug preparation using ampoules/vials	Nasotracheal aspiration	Nasogastric drug administration
SAI total score	−0.065	−0.002	0.059	−0.094	−0.035	−0.170	−0.186	−0.148	−0.064	0.033	−0.130	−0.071
TAI total score	0.024	0.054	−0.092	−0.149	−0.062	−0.113	−0.018	−0.155	−0.022	0.013	−0.182*	−0.166
Hand skill subtests												
Right hand	0.025	0.119	0.135	0.135	0.065	0.020	0.047	−0.043	0.114	0.112	−0.022	0.061
Left hand	0.022	−0.072	0.038	0.092	0.060	0.010	0.033	0.029	0.030	0.002	−0.044	0.197*
Both hand	0.066	0.117	0.133	0.152	0.132	0.108	0.174	0.191*	0.174	0.083	0.034	−0.034
Assembly	0.123	0.134	0.132	0.200*	0.087	0.089	0.277**	0.168	0.164	0.119	0.080	0.168

* *p* < 0.05

** *p* < 0.01.

## 4 Discussion

This study presents the first normative data on the relationship between basic nursing skills, hand function and anxiety level of nursing students. In this respect, the study has a high original value.

The findings of this study suggest that the anxiety levels of nursing students were more moderate compared to those reported in previous research. In particular, the relatively lower levels of trait anxiety may be associated with the structure of the learning environment and the educational strategies employed. Prior studies [29, 30, 31] have emphasised that high levels of anxiety in nursing students are often linked to factors such as lack of clinical experience, fear of causing harm to patients, and the pressure of mastering new procedures. The fact that a majority of students in this study had no prior experience with nursing skills is noteworthy in this context. However, the absence of a significant relationship between anxiety levels and either demographic variables or basic nursing skill performance may indicate that structured practice, effective feedback, and active student engagement helped buffer the potential negative effects of anxiety on psychomotor learning.

In the study, it was determined that among the basic skills performed by nursing students, the lowest mean score was perineal care for women, while the highest mean score was blood collection skill. In a study, 38.8% of nursing students reported that they were afraid of providing perineal care to healthy/patient individuals in clinical practice [32]. Gül et al. [33] stated that the skill that nursing students had the most opportunity to observe during clinical practice was blood collection skill (71.9%). In addition, the ability of the student to obtain immediate feedback (blood in the syringe, etc.) in the blood collection skill in the laboratory is generally a situation that increases motivation. In this respect, the results of the study are consistent with the literature.

When the hand skills of the students participating in the study were evaluated, it was determined that 93.6% (*n* = 117) had the right dominant hand. There is a similar situation in many studies [17, 34, 35, 36]. In the gender‐based comparisons, female students demonstrated significantly higher performance in the left‐hand, both‐hands, and assembly subtests, although no significant difference was observed in the right‐hand subtest. These findings are consistent with previous studies suggesting that female students may exhibit greater fine motor control, particularly in tasks involving bilateral coordination and complex manipulation [12]. Regarding anxiety levels, although mean scores differed, there was no statistically significant difference between female and male students in either the State Anxiety Inventory or the Trait Anxiety Inventory scores (*p* > 0.05). These results suggest that situational anxiety experienced during skill performance was comparable between genders, possibly influenced by the supportive structure of the learning environment.

In the study, the mean score of the right hand skill subtest was 14.92 ± 1.93, the mean score of the left hand skill subtest was 13.76 ± 1.75, the mean score of both hand skill subtest was 11.18 ± 1.66, and the mean score of the assembly subtest was 31.24 ± 6.17. Shetty et al. [37] investigated the effect of smartphone use on manual skills in medical students between the ages of 18 and 27 years. It was reported that 15 ± 2, 14 ± 2, 12 ± 2 and 12 ± 1 for the dominant hand task, 12 ± 2 and 12 ± 1 for the nondominant hand task, and 38 ± 6 and 36 ± 5 pins for the assembly task, respectively, were obtained for both males and females. Kuzgun and Denat [38] defined the manual skills of 196 nursing students with a mean age of 20.7 ± 1.56 years and reported that the mean dominant manual skills score was 19.16 ± 1.36; the mean nondominant manual skills score was 17.04 ± 1.43; the mean both manual skills score was 14.58 ± 1.35; and the mean assembly skills score was 38.55 ± 6.02. Since manual skill is crucial for learning occupational skills in the process of professionalisation in education and labour force participation, it is critical to determine the normative values of fine motor performance across age categories and cultural structure. When compared with the findings of the studies in the literature dealing with similar age groups, it was found that the average skill scores of the participants in this study were lower than the other studies. Although it is difficult to compare the skill scores with the findings of other studies due to methodological differences, this may be interpreted as low hand skill scores due to the fact that the nursing students in the study were still in their first year and that young people had high screen exposure instead of activities aimed at general skill development.

In the findings revealing the relationship between basic nursing skills and manual skills, it was determined that there was a relationship between the basic nursing skills of nasogastric drug administration, blood collection, wearing sterile gloves and subcutaneous injection and manual skills. All of the related skills involve actions such as grasping small objects, manipulation and coordination of sensitive movements. Furthermore, while a significant relationship was found between hand function and some basic nursing skills, the absence of such a relationship in others may be attributed to the varying complexity and motor demands of each skill. Tasks such as subcutaneous injection or blood collection require fine bilateral coordination and sustained hand steadiness, making them more sensitive to variations in manual dexterity compared to more gross‐motor dependent procedures like bed‐making or perineal care. This suggests that the sensitivity of a nursing skill to hand function may differ depending on its nature and required level of precision and coordination.

Although the correlation coefficients identified in this study (e.g., *r* = 0.197, *r* = 0.277) were relatively low, they were statistically significant and provide useful insight into the relationship between hand function and basic nursing skills. In the context of educational and behavioural sciences, small effect sizes are not uncommon and can still have practical relevance, particularly given the multifactorial nature of psychomotor skill development. Manual dexterity is just one of many factors influencing nursing performance. Nonetheless, identifying even a modest association suggests that hand function plays a role worth considering when designing educational interventions. These results support the integration of fine motor skill assessments as a supplementary tool to guide the identification of students who may benefit from additional support in skill acquisition.

The findings of this study are generally consistent with previous research emphasising the role of manual dexterity in the successful execution of clinical nursing procedures. For instance, studies by Kuzgun and Denat [38] and Polat et al. [12] similarly found that higher manual skill levels are associated with more effective technical performance. However, unlike some earlier studies that reported a broader relationship between hand function and a wide range of nursing skills (e.g., [7]), our study identified significant correlations only in specific skills such as blood collection and subcutaneous injection. This partial alignment may be due to differences in sample characteristics, timing of evaluation (first‐year students), or the complexity level of the skills assessed. These findings contribute to the literature by providing a more nuanced understanding of which clinical skills are most influenced by manual dexterity in nursing students.

In addition to professional knowledge, nursing requires care and treatment practices that require a high standard of practical fine motor skills. Students should not have low manual skill for the successful application of basic nursing skills. Especially the low level of these skills seems to be an obstacle to excellence in nursing care. However, when students are placed in nursing programmes, only university exam success scores are decisive [39, 40]. In addition, it has been emphasised in other studies that students can improve their hand skills by receiving vocational training and repetition. Considering the findings of this study, educators should apply hand skill tests in gaining competence starting from basic nursing courses, identify students with low fine motor skills and develop educational programmes‐strategies for these students. Testing the fine motor skills of nursing students with reliable measurement tools such as the Purdue Pegboard Test will also help document the progress gained throughout the education. Such assessments could be integrated into entry‐level skills courses or used during clinical readiness evaluations to tailor individualised training plans.

## 5 Limitations

This study was conducted exclusively with first‐year nursing students from a single university, which may limit the generalisability of the findings. Upper‐year students with greater clinical experience may exhibit different levels of manual dexterity and nursing competencies. Furthermore, the cross‐sectional design of this study prevents the assessment of changes in hand function over time. To address this limitation, longitudinal studies are recommended to explore the development of manual skills throughout the course of nursing education.

Additionally, the study focused solely on hand function, without considering other factors that may influence performance, such as cognitive load, fatigue, motivation, or stress. Finally, the use of a single hand function test may restrict the scope of the findings; employing multiple, validated measurement tools could offer a more comprehensive evaluation of students' manual abilities. While the State‐Trait Anxiety Inventory was used to assess general anxiety, a task‐specific anxiety scale could have offered a more direct evaluation of its impact on performance. Lastly, although one evaluator was used to ensure scoring consistency, the lack of inter‐rater reliability testing is acknowledged as a limitation that may have introduced bias.

## 6 Conclusion and Recommendations

This study offers valuable insights into the relationship between basic nursing skills, hand function, and anxiety levels in nursing students. The results indicate a significant association between hand function and certain basic nursing skills, while no relationship was found between anxiety levels and skill performance. Based on these findings, it is recommended that nursing students' manual abilities be assessed using a multidimensional approach with diverse and validated measurement tools. Competency development should be tracked across academic years, and future research should include larger, more diverse sample groups to improve generalisability. It should also be acknowledged that hand function is only one of several factors influencing clinical competence, alongside cognitive, emotional, and instructional variables.

To improve students' preparedness for clinical practice, nursing curricula should incorporate fine motor skill assessments as a standard component of student evaluation. For example, manual dexterity testing could be implemented during entrance examinations, in early laboratory‐based training, or as part of ongoing competency‐based assessments. In addition, targeted skills laboratory sessions should be organised for students identified with manual dexterity deficiencies, and their progress should be monitored individually over time. Using reliable tools such as the Purdue Pegboard Test can facilitate objective tracking of student development. Looking forward, nursing education accreditation bodies may consider including psychomotor assessment standards in programmatic requirements to support early identification of skill gaps and improve training outcomes. Emphasising manual dexterity in nursing education and implementing structured support strategies will better prepare students for the technical demands of clinical practice, ultimately enhancing the quality of patient care.

## Author Contributions

Study design: Y.C.E., Y.I.A., and S.M. Data collection: Y.C.E. and Y.I.A. Data analysis: Y.C.E. and Y.I.A. Drafting of manuscript: Y.C.E., Y.I.A., and S.M. Critical revisions for important intellectual content: Y.C.E., Y.I.A., and S.M.

## Conflicts of Interest

The authors declare no conflicts of interest.

## Data Availability

The data that support the findings of this study are available from the corresponding author upon reasonable request.

## References

[1] O. Hernon , E. McSharry , I. MacLaren , and P. J. Carr , “The Use of Educational Technology in Teaching and Assessing Clinical Psychomotor Skills in Nursing and Midwifery Education: A State‐of‐the‐Art Literature Review,” Journal of Professional Nursing 45 (2023): 35–50, 10.1016/j.profnurs.2023.01.005. 36889892

[2] Nursing and Midwifery Council , Standards for Preregistration Nursing Programmes (Nursing and Midwifery Council, 2023). https://www.nmc.org.uk/standards/standards-for-nurses/standards-for-preregistration-nursing-programmes/.

[3] S. R. Kemery and B. L. M. Morrell , “Differences in Psychomotor Skills Teaching and Evaluation Practices in Undergraduate Nursing Programs,” Nursing Education Perspectives 41, no. 2 (2020): 83–87, 10.1097/01.NEP.0000000000000515. 31232871

[4] R. Morgan , “Using Clinical Skills Laboratories to Promote Theory–Practice Integration During First Practice Placement: An Irish Perspective,” Journal of Clinical Nursing 15, no. 2 (2006): 155–161, 10.1111/j.1365-2702.2006.01237.x. 16422732

[5] R. Clerkin , D. Patton , Z. Moore , L. Nugent , P. Avsar , and T. O'Connor , “What Is the Impact of Video as a Teaching Method on Achieving Psychomotor Skills in Nursing? A Systematic Review and Meta‐Analysis,” Nurse Education Today 111 (2022): 105280, 10.1016/j.nedt.2022.105280. 35139443

[6] D. Öztürk , N. Çalışkan , Z. G. Baykara , A. Karadağ , and H. Karabulut , “Determining the Effect of Periodic Training on the Basic Psychomotor Skills of Nursing Students,” Nurse Education Today 35, no. 2 (2015): 402–407, 10.1016/j.nedt.2014.10.023. 25466792

[7] C. Reaves , M. Martel , and K. Rose , “Teaching Psychomotor Skills in Undergraduate Nursing Education: An Integrative Review,” Journal of Nursing Education 63, no. 7 (2024): 421–426, 10.3928/01484834-20240505-01. 38979741

[8] Y. Denat and H. Kuzgun , “The Manual Dexterity of Nurses and Factors That Affect It,” Athens Journal of Health & Medical Sciences 7, no. 3 (2020): 145–156.

[9] K. Missen , L. McKenna , and A. Beauchamp , “Registered Nurses' Perceptions of New Nursing Graduates' Clinical Competence: A Systematic Integrative Review,” Nursing & Health Sciences 18, no. 2 (2016): 143–153, 10.1111/nhs.12249. 26592371

[10] A. Michimata , T. Kondo , Y. Suzukamo , M. Chiba , and S. I. Izumi , “The Manual Function Test: Norms for 20‐ to 90‐Yearolds and Effects of Age, Gender, and Hand Dominance on Dexterity,” Tohoku Journal of Experimental Medicine 214 (2008): 257–267, 10.1620/tjem.214.257. 18323695

[11] B. Öztürk , E. Akpinar , R. Akarsu , and Y. Çelik , “Investigation of the Relationship Between Functional Skills, Sensory Functions, and Anthropometric Properties of the Hand in Occupational Therapy Students Using Hierarchical Clustering Analysis,” Ergoterapi ve Rehabilitasyon Dergisi 11, no. 3 (2023): 101–112, 10.30720/ered.1238943.

[12] S. Ö. Polat , E. İ. Işik , E. Tutuş , and P. Göker , “The Effect of Hand Morphometric Measurements on the Manual Dexterity in Students of Vocational School of Health Services,” Cukurova Medical Journal 48, no. 3 (2023): 879–887, 10.17826/cumj.1323882.

[13] I. C. Sigirtmac and C. Oksuz , “Determination of the Optimal Cutoff Values and Validity of the Purdue Pegboard Test,” British Journal of Occupational Therapy 85, no. 1 (2022): 62–67, 10.1177/03080226211008046. 40337111 PMC12033740

[14] R. Causby , L. Reed , M. McDonnell , and S. Hillier , “Use of Objective Psychomotor Tests in Health Professionals,” Perceptual and Motor Skills 118, no. 3 (2014): 765–804, 10.2466/25.27.PMS.118k27w2. 25068745

[15] K. E. Kobayashi‐Cuya , R. Sakurai , N. Sakuma , et al., “Hand Dexterity, Not Handgrip Strength, Is Associated With Executive Function in Japanese Community‐Dwelling Older Adults: A Cross‐Sectional Study,” BMC Geriatrics 18 (2018): 192, 10.1186/s12877-018-0880-6. 30143006 PMC6109297

[16] O. Bezdicek , T. Nikolai , M. Hoskovcová , et al., “Grooved Pegboard Predicates More of Cognitive Than Motor Involvement in Parkinson's Disease,” Assessment 21, no. 6 (2014): 723–730, 10.1177/1073191114524271. 24590077

[17] A. Y. Irmak and A. P. Ketenciler , “Purdue Manual Dexterity Testing: Normative Data From Young People From Turkey,” Journal of Hand Therapy 12 (2025). 10.1016/j.jht.2025.02.012. 40082158

[18] A. Berencsi , F. Gombos , P. Gerván , et al., “Musical Training Improves Fine Motor Function in Adolescents,” Trends in Neuroscience and Education 27 (2022): 100176, 10.1016/j.tine.2022.100176. 35501026

[19] Y. C. Wang , R. W. Bohannon , J. Kapellusch , A. Garg , and R. C. Gershon , “Dexterity as Measured With the 9‐Hole Peg Test (9‐HPT) Across the Age Span,” Journal of Hand Therapy 28, no. 1 (2015): 53–60, 10.1016/j.jht.2014.09.002. 25449717

[20] B. Bakir , N. Kocak , C. Ozcan , T. Kir , M. Cetin , and T. Fedai , “Correlation of Purdue Pegboard Dexterity Test Scores With Class Rank of Turkish Nursing Students,” TAF Preventive Medicine Bulletin 12, no. 6 (2013): 619–624.

[21] Nursing and Midwifery Board of Australia . 2006. National Competency Standards Fort He Registered Nurse. http://www.nursingmidwiferyboard.gov.au/Codes-Guidelines-Statements/Codes-Guidelines.aspx#competencystandards.

[22] J. A. Walker , “Core Clinical Nurse Specialist Practice Competencies.” Clinical Nurse Specialist Role and Practice: An International Perspective (Springer International Publishing, 2021), 37–52.

[23] World Health Organization , Global Strategic Directions for Nursing and Midwifery (World Health Organization, 2021), 2021–2025.

[24] D. M. Romyn , N. Linton , C. Giblin , et al., “Successful Transition of the New Graduate Nurse,” International Journal of Nursing Education Scholarship 6, no. 1 (2009), 10.2202/1548-923X.1802. 19883374

[25] M. T. Hickey , “Preceptor Perceptions of New Graduate Nurse Readiness for Practice,” Journal for Nurses in Staff Development (JNSD) 25, no. 1 (2009): 35–41, 10.1097/NND.0b013e318194b5bb. 19182556

[26] T. L. Hutchinson and H. Janiszewski Goodin , “Nursing Student Anxiety as a Context for Teaching/Learning,” Journal of Holistic Nursing 31 (2013): 19–24, 10.1177/0898010112462067. 23065057

[27] T. N. Vo , H. Y. Chiu , Y. H. Chuang , and H. C. Huang , “Prevalence of Stress and Anxiety Among Nursing Students: A Systematic Review and Meta‐Analysis,” Nurse Educator 48, no. 3 (2022): E90–E95, 10.1097/NNE.0000000000001343. 36538669

[28] N. Öner and A. Le Compte , Handbook of State‐Trait Anxiety Inventory (Boğaziçi University Press, 1983).

[29] M. A. Aloufi , R. J. Jarden , M. F. Gerdtz , and S. Kapp , “Reducing Stress, Anxiety and Depression in Undergraduate Nursing Students: Systematic Review,” Nurse Education Today 102 (2021): 104877, 10.1016/j.nedt.2021.104877. 33905898

[30] B. Öztaş , B. Kara , H. Zengin , A. Güçlü , and B. On , “Simülasyon laboratuvarında verilen eğitimin hemşirelik öğrencilerinin intravenöz kateterizasyon becerisine etkisi,” Hacettepe University Faculty of Nursing Journal 9, no. 1 (2022): 17–23.

[31] A. Cornine , “Reducing Nursing Student Anxiety in the Clinical Setting: An Integrative Review,” Nursing Education Perspectives 41, no. 4 (2020): 229–234, 10.1097/01.NEP.0000000000000633. 32102067

[32] İ. K. Tosunöz , S. Güngör , and G. Öztunç , “Anxiety Before the First Clinical Practice: The Example of Nursing Students,” YOBU Faculty of Health Sciences Journal 2, no. 1 (2021): 14–21.

[33] Ş. Gül , G. H. T. Çelik , S. Arslan , and G. Basit , “Evaluation of Basic Nursing Activities During Clinical Practice of Fundamentals of Nursing: A Retrospective Study,” Health and Society 29, no. 1 (2019): 54–64.

[34] D. T. Hughes , S. J. Forest , R. Foitl , and E. Chao , “Influence of Medical Students' Past Experiences and Innate Dexterity on Suturing Performance,” American Journal of Surgery 208, no. 2 (2014): 302–306, 10.1016/j.amjsurg.2013.12.040. 24815527

[35] S. Özandaç Polat , E. İ. Işık , E. Tutuş , and P. Göker , “The Effect of Hand Morphometric Measurements on the Manual Dexterity in Students of Vocational School of Health Services,” Cukurova Medical Journal 48, no. 3 (2023): 879–887, 10.17826/cumj.1323882.

[36] S. Özandaç Polat , E. İ. Işık , B. Kelle , and P. Göker , “Evaluation of the Association Between Carrying Angle, Hand Grip Strength, Lateral Pinch Strength, and Hand Functional Index in Nursing Students,” International Journal of Morphology 41, no. 5 (2023): 0.

[37] R. Shetty , Y. Gupta , S. Palsule , J. Kale , and P. Shah , “Effect of Smartphone Use on Hand Dexterity in Medical Students: An Observational Cross‐Sectional Study,” Indian Journal of Occupational Therapy 51 (2019): 136–139.

[38] H. Kuzgun and Y. Denat , “The Manual Dexterity of Nursing Students and Factors That Affect It,” International Journal of Occupational Safety and Ergonomics 26, no. 1 (2018): 9–14, 10.1080/10803548.2018.1442909. 29521574

[39] Y. Levanon , D. Lugassy , R. Pilo , et al., “Assessment of the Modified O'Connor Tweezer Dexterity and Purdue Pegboard Test for Use Among Dental Students,” Journal of Dental Education 87, no. 4 (2023): 533–539, 10.1002/jdd.13137. 36374560

[40] M. Giuliani , C. Lajolo , L. Clemente , et al., “Is Manual Dexterity Essential in the Selection of Dental Students?,” British Dental Journal 203, no. 3 (2007): 149–155, 10.1038/bdj.2007.688. 17694029

